# The CASA theory no longer applies to desktop computers

**DOI:** 10.1038/s41598-023-46527-9

**Published:** 2023-11-11

**Authors:** Evelien Heyselaar

**Affiliations:** Communication and Media, Radboud University, Nijmegen, 6525XZ The Netherlands

**Keywords:** Psychology, Human behaviour

## Abstract

The Computers Are Social Actors (CASA) theory is the most important theoretical contribution that has shaped the field of human–computer interaction. The theory states that humans interact with computers as if they are human, and is the cornerstone on which all social human–machine communication (e.g., chatbots, robots, virtual agents) are designed. However, the theory itself dates back to the early 1990s, and, since then, technology and its place in society has evolved and changed drastically. Here we show, via a direct replication of the original study, that participants no longer interact with desktop computers as if they are human. This suggests that the CASA Theory may only work for emergent technology, an important concept that needs to be taken into account when designing and researching human–computer interaction.

## Introduction

The *Computers Are Social Actors* (CASA) theory can be referred to as the most important theoretical contribution to the field of human–machine communication^1^. However, the seminal publication of this theory dates back 30 years; 30 years during which the development, accessibility, and integration of technology in society has changed dramatically. It is therefore not surprising that scholars are revisiting this theory with the aim of updating it to integrate these changes^1–3^. However, these suggestions are based on the assumption that the original CASA theory still holds true, despite the societal changes, an assumption that has, until now, not been investigated empirically.

One main reason why no direct replication has been conducted before now, is that the proposed underlying psychological basis for the CASA Theory suggested that a short period of time (e.g., 30 years) should have no influence on the effect. Specifically, the original authors postulated that the reason we respond to social computers as if they have an awareness is because our brains are not evolving at the same rate as technology; our brains are still adapted to our early ancestors’ environment^4^. In this world, living creatures (and within that group, mostly humans) were the only origins of rich social behaviour. Consequently, psychological mechanisms evolved which allowed automatic and therefore efficient processing of complex social situations resulting in the evolution of rapid, automatic reactions^5,6^. It is only since the 1990’s that a non-living origin of social behaviour has emerged: the computer. As psychological mechanisms are based on thousands of years of evolution, Reeves and Nass claim that it is very unlikely that our brains have developed an automatic way to react differently to social cues from something non-sentient. Hence, if computers send cues which are at least similar to the social cues sent by humans (for example, giving praise for a job well done), these cues will elicit the “ancient” mechanisms originally evolved to efficiently react to other human beings. Hence, we automatically and unconsciously react socially to cues given by a computer. Nass and colleagues argue this theory to be different to the older theory of anthropomorphism^7,8^ as anthropomorphism is based on the conscious belief that the nonhuman entity has human characteristics, whereas the CASA theory is based on the unconscious, social reactions to computers. Reeves and Nass claim that “these [anthropomorphic] responses … require a lot of effort, they tend not to occur when people are tired or when other things compete for their attention, and they are always difficult to sustain. The automatic response [CASA] is to accept what seems to be real as in fact real.” (^4^, p. 8). They suggest that “it is belief, not disbelief, that is automatic”.

An important aspect of the CASA Theory, and why it is still so prominently used today, is that time and experience should not influence the effect: “While it might be tempting to assign the confusion between media and real life to problems of age, knowledge, distraction, or convenience, our research shows that social and natural responses are remarkably common, and true for every group we have tested … *All* people automatically and unconsciously respond socially and naturally to media” (Reeves & Nass, 1997, p. 7).

There have been many indirect replications of the CASA studies since it was first published, however, the results have been mixed. In addition to studies replicating the CASA effect (humans interacting with social technologies as if they were human) with social technology other than a desktop computer^9–13^, studies that found no CASA effect have also been reported^14–17^. Upon closer examination, one potential explanation could be how “emergent” the tested technology is. With emerging technology, you have a novelty effect^18^: That is, technology that people have not had a lot of exposure to are rated more positively than other “older” technology. Unfortunately, after a short period of time, this effect diminishes and the novelty effect wears off. This novelty effect has been shown in robot tutors for children, such that initially the children learn more from the robot than traditional teaching methods, but after three weeks this improvement diminishes^19,20^. A recent study investigated whether we could be friends with a social computer, in which participants were asked to converse with a chatbot over a period of three weeks and constantly rate their relationship. The results showed that initially participants were enthusiastic and engaging with their chatbot friend, but quickly this diminished, with scores for intimacy, believability, and likability decreasing with each interaction^21^. Hence it is possible that the novelty effect caused the highly positive ratings reported in the original CASA experiments.

In the early 1990s, when the data for the original study was collected, there was no Internet available in the home. In fact, by the turn of the twentieth century, only one third of households in the UK owned a home PC^22^, or had access to the Internet^23^. Nass and Reeves make a point of stating in their methodology that all the participants “have extensive experience with computers … they were all daily users, and many even did their own programming” (*4,* p. 26). In^24^, this “daily use” is defined as having “extensive e-mail and word-processing experience” (p. 1097). Taken together, even though these participants can work a computer, they lived in a society where Internet access was not common. Most, if not all, of their interactions with a computer was composing text via a word-processing service. They were not accustomed to a computer showing any sort of social behaviour, whether from the computer “itself” or from another human via a chatroom or similar. Therefore, having any kind of social interaction with a computer was highly novel at the time the original studies were conducted.

Just as the original participants did not have a lot of experience interacting with social computers, the same goes for indirect replications that showed the CASA effect. For example, having participants interact with a voice-activated navigation system in 2005^12^, looking into reciprocity effects with the virtual assistant Alexa^13^, or having participants being asked for help by a lost robot in 2016^25^. To compare, when the technology is no longer novel, the CASA effect was not observed: e.g., with a text-based chatbot in 2003^17^ or robots in 2021^14^. What we have referred to here as *indirect* replications, could also easily be viewed as *conceptual* replications: replications using the same methodology but using technology that was equally novel at the time of testing as the desktop computer was for the original CASA participants.

The current study is the first direct, non-conceptual replication of the seminal CASA study published in 1994: Are humans polite to computers^26^. Like the original study, we investigated this phenomenon by having participants rate the performance of a desktop computer either directly, or via an identical desktop computer in another room. The design is based on the social phenomenon that an interviewer who directly asks about themselves will receive more positive responses than if the same questions were posed by a third party. The original study showed that participants (N = 10 per condition) were more positive about the performance of a computer tutor if the evaluation was conducted on that same computer (Same Computer condition), compared to if the evaluation was conducted on an identical, but different, computer in another room (Different Computer condition).

We purposefully used a simple desktop computer. If the CASA effect does occur automatically and always, as the original authors claim and as contemporary researchers assume, we should observe the CASA effect. If we do not, it suggests that older technology, such as the desktop computer, is no longer viewed as a social actor. If this is the case, it would mean that current emergent social technology (e.g., chatbots, robots, virtual agents) will also not been seen as social actors in the near future. This is an important aspect that needs to be investigated.

## Results

**Figure 1:**
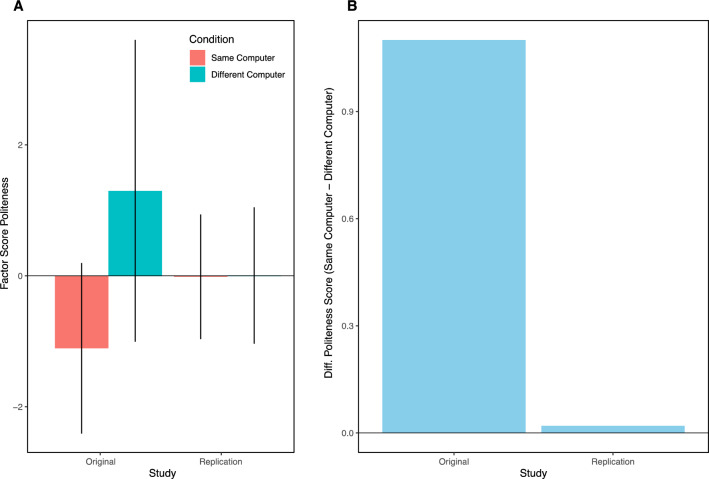
Difference in performance between the original 1994 study and the 2022 direct replication. (**A**) Shows the factor scores for each condition, for each study. Error bars represent standard deviation (**B**) shows the differences in factor scores (Same Computer—Different Computer).

The original study (with 20 participants) reported a large difference in the positivity of the evaluation (Raw Index/10: Same Computer = 5.6; Different Computer = 4.5, two-sample *t*(17) = 3.5, *p* < 0.001, omega-squared = 0.36). With our direct replication of 132 participants, we were not able to replicate this large difference (Raw Index/10: Same Computer = 5.7; Different Computer = 5.7, two-sample *t*(130) = − 0.2, *p* = 0.852, Cohen’s *d* = − 0.03). Figure 1 illustrates these differences.

The original study additionally investigated the homogeneity of the answers, arguing that greater variance in responses has been associated with greater honesty. The authors therefore predicted to find a *smaller* variance in the Same Computer condition compared to the Different Computer condition, predicting that the participants will be more polite in the Same Computer condition, and therefore less honest (a so-called “white lie”). Indeed, this significant difference was found in the original study (Mean heterogeneity of items: Same Computer = 2.0; Different Computer = 2.5, *t*(20) = 3.3, *p* < 0.001). We conducted the same analysis with our data, with the prediction that we should find no significant difference, as our participants see no need to be more or less honest towards the computer. A computer does not have feelings or emotions and hence would not benefit from the participant producing “white lies” (dishonesty in the form of a more positive review). Indeed, in our direct replication we found no difference in the homogeneity of the answers (Mean heterogeneity of items: Same Computer = 2.2; Different Computer = 2.1, *t*(40) = 0.6, *p* = 0.533).

## Discussion

The current study is the first direct replication of the original CASA study: Are humans polite to computers. We showed no CASA effect: Participants did not feel the unconscious, automatic belief that the desktop computer would benefit from them being “dishonest” by being more polite in reviewing the computers performance. This has far-reaching implications.

The CASA Theory has had a profound impact in multiple fields, such as psychology, social sciences, communication science, and artificial intelligence, to name but a few. The work of Clifford Nass and his team has become the foundation on which social agents have been developed. As developers have assumed that users interact with social computers as if they are human, social computers have been more easily integrated into commercial applications to support human jobs. For example, as robot tutors for children^27^, in the form of chatbots to help with online inquiries^28^, and virtual therapists as a first round of diagnoses^29^. It would be fair to state that the majority of human-agent interaction research and application is based on the CASA Theory. Currently, these applications work as the technology is emergent enough to exhibit a CASA effect from the users. However, given the current data, it is possible that in a single generation, these applications are no longer viable.

We claim that our replication is a direct replication, which was made possible by the experimenter scripts and the content available in the appendices of the original studies. We intricately followed the methodology published in these studies, such as ensuring that “every effort was made to reduce social demand characteristics. Neither pictures of people or characters nor audio cues were employed. The computer referred to itself as “this computer” rather than “I”, referred to the participant as “you” rather than with a name, and the computer never used language that suggested a “self” or “emotions”” (p. 1098). It was this procedure that made the original findings so impressive: the experiment itself tries to *not* trigger human-like associations with the desktop computer. However, it seems that in the early 1990s, participants were more open to the possibility of agency in computers compared to today.

The aim of the current study was not to conduct a *conceptual* replication: having participants in 2022 interact with something they view as highly novel. The aim of the current study was to have the participants in 2022 directly repeat the experiment as it was conducted in the 1990’s. As the original CASA Theory states that experience should not influence the effect, if this were true, we should see a CASA effect even with “older” technology. Yet, our results indicate that the CASA theory *doesn’t* apply to “older” technology.

There are other factors than simply the aging of technology that could have influenced our results. Although we used a simple desktop computer, we were not able to acquire the actual model used in the original experiments. Additionally, due to the Internet of Things, it is sometimes unconsciously assumed that everything is connected via the Cloud. Hence, participants may have assumed that the Same Computer has access to the evaluations conducted on the Different Computer, and that participants were actually polite in both conditions. Follow-up studies explicitly measuring these potential moderators will need to be conducted. Additionally, the CASA theory is based on many other social phenomena, not just politeness, therefore, direct replications of the other CASA studies will help shed light on the different aspects that could be effecting this change.

Here we show for the first time that the CASA effect is short-lived. In our study, there was a difference of 30 years (one generation), however, the effect may have gone much earlier. The integration of social human–computer interaction into our daily lives has changed our societal landscape, and hence it is not surprising that this, in turn, changes the way we interact with technology. Our current study is the first in investigating this delicate balance.

## Materials and methods

### Participants

The original study recruited 30 Stanford University undergraduates (10 in each of the original 3 conditions, see *Procedure*). Following a power-analysis for a two-tailed two-sample *t* test, assuming a medium effect size (d = 0.5; alpha = 0.05), for a power of 0.8, G*Power (3.1) recommended a total sample size of 128 (64 in each condition). Our eventual sample consisted of 132 participants (75.7% female, M_Age_: 22.7 years, SD_Age_: 2.9 years) recruited from the Radboud University Experiment Database.

Participants were given €5 for participating or 0.5 credits (if participating as part of a course requirement). The research has a minimal risk and was approved by the Ethics Committee for Social Sciences (ECSS) of the Radboud University (ECSW-LT-2022-3-11-90198). All methods were performed in accordance with the guidelines and regulations as set up by the ECSS. Before the experiment, active informed consent was obtained from all participants.

### Procedure

The procedure is based on that reported in^30^, except that for the current study we only included 2 conditions. The original study included a third condition, a paper-and-pencil condition. However, the original paper already argues that differences may have occurred simply due to the modality difference, and hence we decided to exclude this condition entirely in our replication.

Participants were pseudo-randomly assigned to one of the two conditions in this between-subjects design. The Same Computer condition consisted of 74 participants and the Different Computer condition consisted of 58 participants. Re-running the analysis with an equal number in both conditions does not influence the conclusions.

Upon arrival, the participant was told that they would be working a desktop computer to complete a computer-based, interactive tutoring, testing, and scoring session. The details of the tutoring, testing, and scoring session were described to the participants, using the script provided in^30^. Participants were told that after the scoring session, they would be asked to respond to a questionnaire. The researcher left the room when the experiment started.

The procedure described below are exactly as those described in^30^:

#### Tutoring session

Participants were told by the experimenter that the tutoring session was designed to help them learn by selecting facts that best complemented their current knowledge, During the session, the computer presented each participant with 20 facts on the topic of American culture (e.g., “Forty-three percent of American teenagers kiss on the first date”). After being presented with each fact, participants indicated on a 3-point scale, ranging from 1 (*know very little*) to 3 (*know a great deal*), how much they knew about that fact.

Participants were told that they would receive 20 facts out of a total of 1000 possible facts. The participants were told that the facts would be chosen based on the principle that the more familiar they were with a fact, the fewer additional facts they would receive on the same topic. The purpose of this statement was to ensure that the subjects felt that they were interacting with the computer, rather than simply being passives readers. In actuality, all participants received the same 20 facts in the same order.

#### Testing session

After completing the tutoring session, the computer administered a 12-item, five-alternative multiple-choice test. The instructions indicated that a total of 12 questions would be randomly chosen from a set of 5000 questions. In fact, all participants received the same 12 questions (e.g., “What percentage of Americans marry their high school prom date?”). Participants were led to believe that each question had an objectively correct answer. In fact, this was not true.

#### Scoring session

In the instructions, participants were told that the computer would evaluate their performance on the test and its own performance as a tutor. During the scoring session, the computer reviewed each question separately. For each item, the computer indicated whether the participant had given the correct answer and then praised its own performance. For example, “your answer to the question concerning teenagers and rock music was correct. The computer provided very useful facts for answering this question. Therefore, this computer performed extremely well.” The purpose of the computer’s praise of itself was to ensure that the socially desirable response was unambiguously positive. All participants were informed that they had answered the same 8 of the 12 questions correctly; all participants received identical evaluations from the computer.

Every effort was made to reduce social demand characteristics. Neither pictures of people or characters nor audio cues were used. The computer referred to itself as “this computer” rather than “I”, referred to the participant as “you” rather than with a name, and the computer never used language that suggested a “self” or “emotions”.

#### Interview questionnaire

Following the completion of the scoring session, the participant was asked questions about the performance of the computer. Participants responded by typing on the keyboard.

### Manipulation

In the same-computer condition, the interview questionnaire was conducted on the same computer that the participant had worked with during the task (see “Materials and methods”). In the different-computer condition, the interview questionnaire was conducted on a different (but physically identical) computer in the next room. The instructions and questions in each interview condition were identical.

The visual characteristics of the interface (e.g., font, font size, font style, button and text layout) were identical across the two computer conditions.

### Materials

The experiment was presented in a fully enclosed room (“cubicles”). The cubicles are designed for single-person experiments and only feature the essentials for basic computer tasks: a desk, a chair, and a standard lab computer setup. The experiment was run on a Dell Precision T-series computer (Dell Precision 3640), fitted with a 24″ Benq screen (Benq XL2420Z). The experiment was written and run from Presentation (Neuro Behavioural System, version 23.0). For the Different Computer condition, the Presentation script instructed the participant to go into the neighboring lab room (which was fitted with the same equipment) to complete the questionnaire.

### Data analysis

The interview questionnaire consisted of two sets of questions. The first set was initiated with the statement “For each of the following adjectives, please indicate how well it describes the tutoring session.” Participants then indicated how well each of a list of adjectives described the tutoring session: analytical, competent, enjoyable, friendly, fun, helpful, informative, knowledgeable, likable, polite, useful, and warm. For all items, participants answered on a 9-point scale ranging from 1 (*describes very poorly)* to 9 (*describes very well*). The second set of questions was initiated with the statement “For each of the following adjectives, please indicate how well it describes the scoring session,” followed by a second list of adjectives associated with the same 9-point scale: accurate, analytical, competent, fair, friendly, fun, likable, polite, and warm.

Following the analysis procedure as described in the original study, all 21 items were combined into a single factor to capture the participants’ overall valence toward the computer. Higher scores on the factor indicated more positive responses toward the computer. The factor scores for the two conditions were compared using a two-sample *t* test in R (version 4.1.0), using the t.test function from the stats package (version 4.1.0). The data was normally distributed (*p* = 0.513), as tested using the levene_test function from the rstatix package (version 0.7.0).

Following the analysis procedure as described in the original study, for testing the relative homogeneity (dishonesty) of the assessments, we used item analysis. Specifically, for each item, we computed the standard deviation of the item across participants in a given condition. We then treated item as the unit of analysis in a paired-comparison *t* test.

## Data Availability

Experiment and analysis scripts, as well as anonymized data, is available here: 10.17605/OSF.IO/ZN4JS.
